# Clindamycin-Loaded Nanosized Calcium Phosphates Powders as a Carrier of Active Substances

**DOI:** 10.3390/nano13091469

**Published:** 2023-04-25

**Authors:** Dagmara Słota, Karina Piętak, Wioletta Florkiewicz, Josef Jampílek, Agnieszka Tomala, Mateusz M. Urbaniak, Agata Tomaszewska, Karolina Rudnicka, Agnieszka Sobczak-Kupiec

**Affiliations:** 1 Department of Materials Engineering, Faculty of Materials Engineering and Physics, Cracow University of Technology, 37 Jana Pawła II Av., 31 864 Krakow, Poland; 2 Department of Analytical Chemistry, Faculty of Natural Sciences, Comenius University, Ilkovičova 6, 842 15 Bratislava, Slovakia; 3 Department of Chemical Biology, Faculty of Science, Palacky University Olomouc, Slechtitelu 27, 783 71 Olomouc, Czech Republic; 4 Department of Immunology and Infectious Biology, Faculty of Biology and Environmental Protection, University of Łódź, 90-237 Łódź, Poland; 5 Bio-Med-Chem Doctoral School, University of Lodz and Lodz Institutes of the Polish Academy of Sciences, 90-237 Łódź, Poland

**Keywords:** calcium phosphates, ceramics, hydroxyapatite, brushite, drug delivery system, antibiotic, clindamycin

## Abstract

Bioactive calcium phosphate ceramics (CaPs) are one of the building components of the inorganic part of bones. Synthetic CaPs are frequently used as materials for filling bone defects in the form of pastes or composites; however, their porous structure allows modification with active substances and, thus, subsequent use as a drug carrier for the controlled release of active substances. In this study, four different ceramic powders were compared: commercial hydroxyapatite (HA), TCP, brushite, as well as HA obtained by wet precipitation methods. The ceramic powders were subjected to physicochemical analysis, including FTIR, XRD, and determination of Ca/P molar ratio or porosity. These techniques confirmed that the materials were phase-pure, and the molar ratios of calcium and phosphorus elements were in accordance with the literature. This confirmed the validity of the selected synthesis methods. CaPs were then modified with the antibiotic clindamycin. Drug release was determined on HPLC, and antimicrobial properties were tested against *Staphylococcus aureus*. The specific surface area of the ceramic has been demonstrated to be a factor in drug release efficiency.

## 1. Introduction

**Figure 1:**
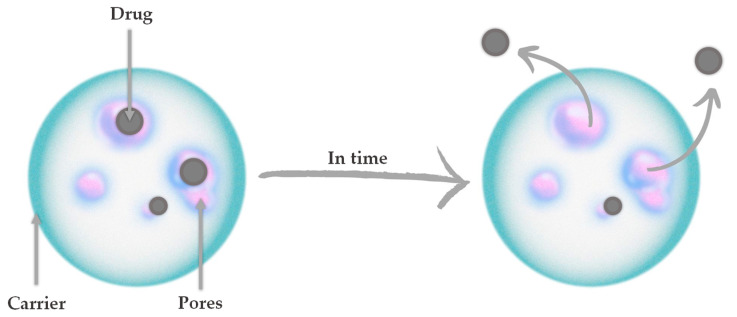
The principle of ceramic drug carrier operation.

The ability to achieve targeted therapy as well as sustained and controlled release of the drug is possible through the use of drug delivery systems (DDSs). It is a hot spot in the medical field understood as a combination of drug and carrier, where the drug is loaded inside or on the surface of the carrier by chemical or physical methods [1]. In general, drug carriers are understood as safe tools for transporting molecules for nutraceutical, pharmaceutical, and cosmetic applications [2]. In recent years, many inorganic nanomaterials such as gold nanoparticles [3,4,5], carbon nanotubes [6,7,8], and quantum dots [9,10,11] have been extensively studied for drug delivery. In the context of bone tissue regeneration, ceramic carriers based on bioactive calcium phosphates (CaPs) are of great interest. One of the advantages of ceramic carriers is their low toxicity. Most CaPs have good biocompatibility, biodegradability, as well as biological stability [12]. Furthermore, the variety of ceramic materials as well as the methods of their synthesis make it possible to adjust the size and structure of the grains. It is therefore possible to adjust these parameters for drug loading, for example, in the nanometer size [13]. Figure 1 presents the principle of ceramic drug carrier operation. The diffusion of drugs and other active substances through the pores of ceramics depends on their concentration gradient and solubility. The porosity of the ceramic carrier itself, as well as the specific surface area and size, affect drug diffusion [14,15].

The existence of CaPs in bones was discovered in 1769, and since 1900 active research has been carried out to synthesize them for medical applications such as bone defect fillers, apatite pastes, and composite materials for implants [16,17,18,19]. In the structure of CaPs are present PO_4_^3−^ and HPO_4_^2−^ ions found in bone and tooth-building minerals, and H_2_PO_4_^−^ ions, which are formed only in acidic reaction environments. They are mostly poorly water-soluble salts of the tri-basic orthophosphoric acid H_3_PO_4_ and dissolve well in acids [20]. There are several types of CaP, which differ in the molar ratio of calcium and phosphorus (Ca/P). This also influences the properties of these ceramic materials. A lower Ca/P ratio corresponds to more acidic and relatively water-soluble phases [21]. Calcium phosphates occur in the CaO–P_2_O_5_ arrangement or, if they contain OH^-^ ions, in the arrangement CaO–P_2_O_5_–H_2_O. Table 1 summarizes synthetic CaPs relevant to medical applications, along with their names, Ca/P molar ratio, and chemical formulae [22,23].

Currently, hydroxyapatite (HA) and tricalcium phosphate (TCP) are widely used as implant materials [24,25]. HA is the most well-known and widespread phase and has a Ca/P ratio of 1.67. It represents the mineral part of natural bone, which consists of 70% of just this inorganic material [26]. TCP has a Ca/P ratio of 1.5 and occurs in two polymorphic varieties (high-temperature α-TCP and low-temperature β-TCP). α-TCP is produced at temperatures above 1125 °C, while β-TCP is formed at temperatures below 1125 °C [27]. In contrast to α-TCP, β-TCP is thermodynamically stable in biological environments and within the normal temperature range. α-TCP exhibits better and faster solubility than β-TCP, and for this reason, the low-temperature variety has found wider application in medicine and dentistry [28,29]. Furthermore, also of interest in the context of applications where the implant is to be replaced by newly formed bone over time is brushite (DCPD). The main difference between DCPD and HA is its solubility in body fluids, in which DCPD is 100 times more soluble than HA. For this reason, brushite biomaterials are resorbable in vivo and will start faster, and this aspect also affects the increase in porosity of such a biomaterial over time, which allows surrounding tissues to grow into it [30,31]. An undeniable advantage of CaPs in the context of their use as biomaterials, including drug carriers, is their biocompatibility, as they do not induce inflammation or other negative reactions. Moreover, some of them exhibit bioactivity (e.g., HA), affecting the proliferation and adhesion of bone-forming cells—osteoblasts [32,33,34]. The bioactivity of ceramics is related to the processes of degradation and release of ions from the material. As a result of this phenomenon, the local concentration of Ca and P ions increases, which stimulates the formation of new apatite layers and bone minerals on the surface of the material. They also affect the expression of osteoblast differentiation markers, such as BMPs, COL1, ALP, BSP, and OCN [29,30,31,32]. The interaction between ceramics and osteoblasts depends on the geometry of the material and is improved if the surface of the phosphate–calcium is charged or polarized. Therefore, for the successful application of the materials, attention should also be paid to their resistance to both ultraviolet and X-rays. The application of the materials depends on their resistance to aging, including but not limited to radiation [35,36,37,38,39]. X-rays promote the decarboxylation of the collagen side chain, which in turn negatively affects the electrostatic bonding between the phosphate groups in CaP and the carboxylate groups of the protein side chains [40].

In the following work, four calcium phosphate ceramic powders were modified with clindamycin using the physical sorption method. Clindamycin is an antibiotic of the lincosamide group [41]. It has a broad spectrum of activity that includes both Gram-positive bacteria (including many strains of MRSA) and anaerobic bacteria. The function of this drug antibiotic is to block the synthesis of bacterial proteins [42]. Clindamycin is most often used in complaints of bacterial infection of the oral cavity or teeth or bacterial infection of bones [43,44]. Hence, the authors decided to select this specific drug for ceramic phase modification in this study. Considering the properties of this drug, it is significant that the authors have also previously used it to modify hydroxyapatite-reinforced hydrogel materials [45]. In this study, using high-performance liquid chromatography (HPLC), the drug release rate was determined, and the antimicrobial properties of the carriers against *Staphylococcus aureus* ATCC 29213 were determined. The clean powders were subjected to physicochemical analysis, including X-Ray diffraction analysis (XRD), Fourier-transform infrared spectroscopy (FTIR), or Ca/P molar ratio determination. No other literature describing identical ceramic-drug combinations was found.

## 2. Materials and Methods

### 2.1. Materials

All reagents used for ceramics s-1.67 and 1.5 syntheses, i.e., the sodium phosphate dibasic (Na_2_HPO_4_), calcium acetate monohydrate (Ca(CH_3_CO_2_)_2_ ∙ H_2_O), and ammonia water (NH_4_OH, 25%) were purchased from Sigma-Aldrich (Darmstadt, Germany). The commercial hydroxyapatite identified in the publication as c-1.67 was also from Sigma-Aldrich (Darmstadt, Germany). The reagents for the brushite ceramics, i.e., disodium hydrogen phosphate dihydrate (Na_2_HPO_4_ ∙ 2H_2_O) and calcium nitrate tetrahydrate (Ca(NO_3_)_2_ ∙ 4H_2_O), were purchased from Sigma-Aldrich (Darmstadt, Germany). The mobile phase in the HPLC was a combination of acetonitrile from Honeywell (Seelze, Germany) and KH_2_PO_4_ from DOR-CHEM (Krakow, Poland). The reagents necessary for the determination of the Ca/P molar ratio were HNO_3_ and HCl from Stanlab (Lublin, Poland), Bi(NO_3_)_3_, triethanolamine, as well as KOH from Sigma-Aldrich (Darmstadt, Germany), and disodium edetate from Warchem (Warsaw, Poland). The drug selected for powder modification was clindamycin hydrochloride from Sigma-Aldrich (Darmstadt, Germany). Demineralized water purified with Hydrolab model HLP 5sp was used for all solutions.

For biological research, the bacteria *Staphylococcus aureus* ATCC 29213 (American Type Culture Collection, Manassas, VA, USA) was purchased. Bacteria were cultured in Mueller–Hinton Broth from Merck (Darmstadt, Germany). Resazurin was from Merck (Darmstadt, Germany), and phosphate-buffered saline (PBS) was from Oxoid (Basingstoke, UK).

### 2.2. Ceramic Synthesis

Four nanopowders were selected for this study. Three of them were obtained by wet precipitation methods with different Ca/P molar ratios in the range of 1.0–1.67, and a commercial hydroxyapatite designated as c-167 was selected as a reference powder. The material synthesis methods are described below.

The hydroxyapatite-structured powder, designated as s-1.67, was synthesized by wet precipitation at boiling temperature. Firstly, solutions of Na_2_HPO_4_ (0.32 mol/L) and (CH_3_COO)_2_Ca (0.128 mol/L) were prepared. Distilled water and a specified volume of Na_2_HPO_4_ were poured into a three-necked flask. Then, using 25% ammonia water, the pH of the system was brought to 11. After the entire system was brought to the boiling point of the ingredients, (CH_3_COO)_2_Ca was dropped in at a rate of 1 drop/sec. After the synthesis was completed, the ceramic suspension was cooled and allowed to stand for 24 h. After this time, the precipitate was washed thoroughly with distilled water, brought to neutral pH, and freeze-dried. This method was also described in a previous paper [46,47].

The ceramic powder, marked as 1.5, was obtained in an analogous manner to s-1.67 by reducing the volume of the added acetate salt accordingly. The ratios of salt solutions were calculated to obtain a Ca/P ratio of 1.5.

The brushite powder, designated as 1.0, was synthesized using the wet precipitation method. In the first step, 500 mL each of aqueous solutions of Na_2_HPO_4_ ∙ 2H_2_O and Ca(NO_3_)_2_∙ 4H_2_O were prepared, where the concentration of both solutions was 0.5 mol/L. The Na_2_HPO_4_ ∙ 2H_2_O solution was placed on a magnetic stirrer, and the prepared Ca(NO_3_)_2_∙ 4H_2_O solution was dropped in at a rate of 1 drop per second. The pH of the solution was maintained between 6 and 6.5 with 25% ammonia water. The resulting ceramic suspension was aged for 24 h, washed thoroughly with distilled water to a neutral pH, and freeze-dried.

### 2.3. Determination of Calcium and Phosphorus Content

The molar ratio of Ca and P (Ca/P) plays a crucial role in the formation of the calcium phosphate phase. Furthermore, the value of the ratio is one of the indicators suggesting which ceramic material the powder under examination belongs to [48]. The determinations were conducted in accordance with the Polish standard for phosphorus based on PN-80/C-87015 for calcium based on PN-97/R-64803 [49,50].

Phosphorus content (wt. %) was determined according to Formula (1):

(1) $$\%P=\frac{\left(\frac{M_{1}\cdot V_{1}\cdot 100\%}{m\cdot V_{2}\cdot 1000}\right)}{2.29}$$

where *M*_1_ is a P_2_O_5_ content in the analyzed sample determined from the standard curve [mg/mL]; *V*_1_—volume of volumetric flask used for phosphorus extraction [mL]; *m*—sample mass used for extraction [g]; *V*_2_—volume of solution collected for analysis [mL]; and 2.29—conversion factor from P_2_O_5_ to P.

Calcium content (wt. %) was determined according to Formula (2):

(2) $$\%Ca=\left(\frac{0.04008\cdot V_{1}\cdot c\cdot V_{2}}{m\cdot V_{3}}\right)\cdot 100$$

where *V*_1_ is a volume of EDTA solution consumed during calcium titration [mL].; *c*—titer of EDTA solution set to calcium standard solution [mol/L]; *V*_2_—volume of sample solution [mL]; *m*—sample mass used for extraction [g]; and *V*_3_—volume of solution collected for calcium titration [mL].

Calcium and phosphorus determinations were repeated for all powders. Each measurement was performed in triplicate.

### 2.4. X-ray Diffraction Analysis

In order to perform structural characterization of the ceramic powders obtained, X-ray diffraction analysis was performed using a Malvern Panalytical Aeris X-ray diffractometer with PIXcel1D-Medipix3 detector (Malvern, UK) with Cu Kα radiation. All measurements were carried out at a step size of 0.0027166° 2θ over a range of 2θ from 10 to 100° with a time per step of 340.425 s.

### 2.5. Fourier-Transform Infrared Spectroscopy Analysis

Individual functional groups in ceramic powders were identified using Fourier-transform infrared spectroscopy (FT-IR). The analysis was carried out under room conditions using a Nicolet iS5 91 FT-IR spectrometer equipped with an iD7 ATR attachment (Thermo Scientific, Loughborough, UK) in the range 4000–400 cm^−1^ (32 scans at 4.0 cm^−1^ resolution).

### 2.6. Specific Surface Area and Porosity Studies

Specific surface area (SSA) and porosity studies were performed using Auto-sorb-1 Quantachrome flow apparatus, with nitrogen as an adsorbate, at −196 °C. Prior to the measurements, all samples were preheated and degassed under vacuum at 200 °C for 18 h. The specific surface area of the samples was determined with the multipoint Brunauer–Emmett–Teller (BET) analysis method. The Brunauer–Emmett–Teller (BET) theory explains the physical adsorption–desorption of gas molecules on a solid surface and represents the basis for the analysis of measured data. Micropore pore volume and micropore surface were determined with the t-plot method, while the mesopore pore volume and mesopore surface were determined with the Barret–Joyner–Halenda (BJH) and density-functional theory (DFT) methods. DFT methods allow one to obtain reliable pore size distributions over the complete range of micro- and mesopores (for a recent review on the application of DFT methods for pore size analysis). Methods for pore size analysis based on DFT and molecular simulation are now widely used and are commercially available for many important adsorptive or adsorbent systems and are featured in a standard by the International Organization for Standardization (ISO). The calculation of the pore size distribution function f(W) is based on a solution of the general adsorption isotherm (GAI) equation, which correlates the experimental adsorption isotherm N(p/p 0) with the kernel of the theoretical adsorption or desorption isotherms N(p/p 0,W).

### 2.7. Preparation of Ceramic Powders with Drug

The antibiotic selected for powder modification by physical sorption was clindamycin hydrochloride. Ceramic weights of 0.5 g each were inserted into 40 mL of the drug solution at a concentration of 2 mg/mL for 5 days. The samples were stored in tightly closed containers at 4 °C. After this time, the samples were centrifuged with a laboratory centrifuge (MPW Med. Instruments MPW—260R) for 15 min, at 4 °C with a speed of 5500 rpm, and then the solution was decanted from the precipitate. The remaining powders were dried and submitted for release kinetics studies. Samples of ceramic powders after drug modification were similarly marked as C/1.0, C/1.5, C/c-1.67, and C/s-1.67.

### 2.8. Kinetic Release Studies of Antibiotic

**Figure 2:**
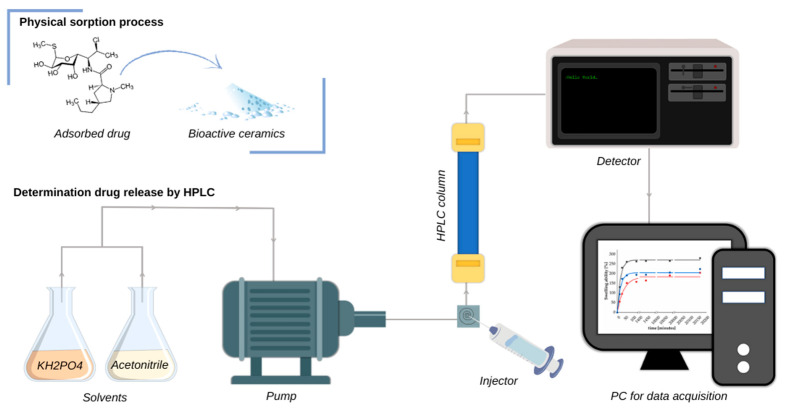
Principle of HPLC for the determination of clindamycin.

The prepared drug-modified ceramic powders were submitted for release kinetics studies. Powder samples of 0.5 g each were incubated in 15 mL PBS over a period of 14 days. To examine the amount of clindamycin released from the samples, the collected incubation fluids were analyzed by high-performance liquid chromatography (HPLC). The principle of HPLC for the determination of released clindamycin is schematically presented in Figure 2.

Determination of drug released from the powders was carried out on a Shimadzu HPLC system (Kyoto, Japan) consisting of an SPD-M20A UV-Vis photodiode array detector, a CTO-20AC column oven, and an LC-20AD pump. The antibiotic was separated on a C18 reversed-phase Kinetex^®^ column (250 mm × 4.6 mm) with a pore size of 100 Å and a particle size of 5 μm. The system was operated isocratically with a mobile phase consisting of a mixture of acetonitrile and KH_2_PO_4_ (45:55 *v*/*v*) at pH = 7.5 at a flow rate of 1.0 mL/min (30 °C). The sample was injected through a 20 μL sampling loop. The experiment was conducted with the detector set at 210 nm. Each time, prior to measurement, 1 mL of the collected incubation fluid was centrifuged at 18,000 rpm at 4 °C using an MPW-260R (Warsaw, Poland).

### 2.9. Morphology Analysis

Scanning electron microscopy (SEM) imaging was performed using a Jeol 5510LV with an EDS IXRF system detector (Freising, Germany) to determine the structure of the powders and their shape. The powders were attached to a carbon tape and then coated with nano-gold. EDS microanalysis was also performed for clindamycin-modified C/s-1.67, determining elements such as Au, Ca, P, and Cl.

### 2.10. Antimicrobial Activity of Clindamycin-Modified Ceramic Powders

The antimicrobial activity of clindamycin-modified ceramic powders against *Staphylococcus aureus* ATCC 29213 was assessed by the determination of the minimum inhibitory concentration (MIC) by classic broth microdilution and resazurin reduction assay [51]. The bacteria were cultured in Mueller–Hinton Broth to mid-log phase, and the inoculum was standardized to 0.5 McFarland (5 × 10^5^ CFU/mL) as recommended by EUCAST guidelines [52]. The clindamycin-modified ceramic powders were distributed into wells of a 96-well plate (Nunc, Rochester, NY, USA) containing 100 μL Mueller–Hinton Broth (MHB) to form a series of 2-fold dilution. Then the bacterial suspension (100 μL) was added to each well, and plates were incubated for 24 h at 37 °C. Control wells contained bacterial culture alone (positive control of bacterial growth), and wells with MHB alone (negative control) were included. In addition, a reference antibiotic, clindamycin, in the range of 256–0.001 µg/mL was included. Four independent experiments were performed in triplicate. Then in the classic method, the MIC was read manually as the lowest concentration of antimicrobial agent that completely inhibits the growth of the organism as detected by the unaided eye. Whereas to assess the MIC_99_ on the basis of microbial metabolic activity prior to reading, the 20 μL of 0.02% resazurin in sterile PBS was added to each well and left for 3 h. Fluorescence was measured at an excitation wavelength of 560 nm and emission wavelength of 590 nm using a SpectraMax^®^ i3x Multi-Mode Microplate Reader (Molecular Devices, San Jose, CA, USA).

## 3. Results

### 3.1. Determination of Calcium and Phosphorus Content

The Ca/P molar ratios for the powders and the determined elemental contents of calcium and phosphorus (wt. %) are presented in Table 2. The determined Ca/P molar ratios differ slightly from the assumed values. In the case of s-1.67 and c-1.67, the values obtained are very close to the stoichiometric HA, which is assigned a value of 1.67. The slight discrepancies that can be observed for all synthesized samples may be due to the incorporation of foreign cations or anions into the atomic structure of the ceramics. The reactions were carried out in an air atmosphere; hence, substitutions of, for example, phosphate groups by CO_3_^2-^ ions are possible.

### 3.2. X-ray Diffraction Analysis

**Figure 3:**
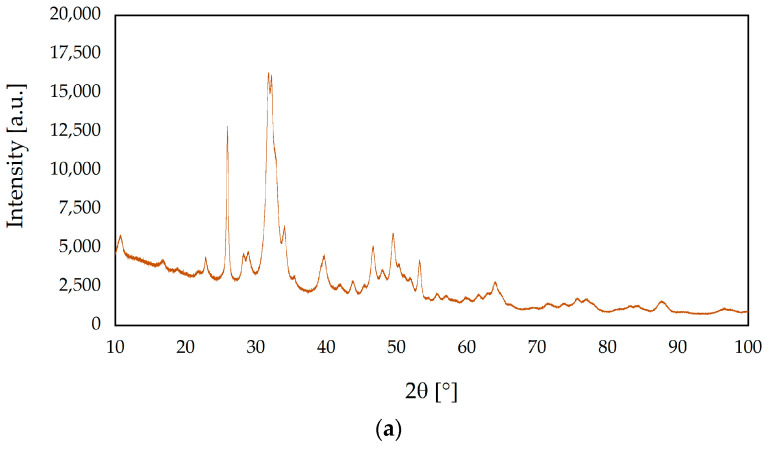

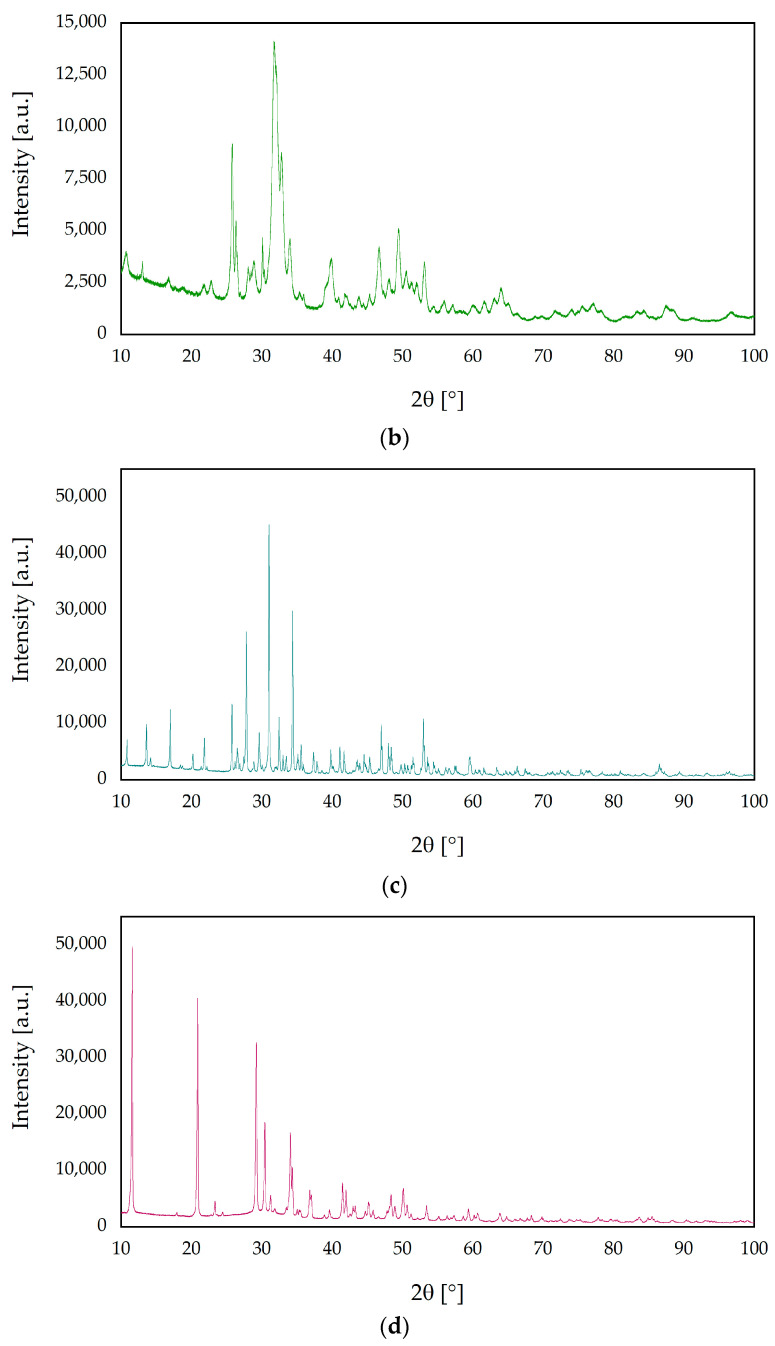
X-ray diffraction (XRD) patterns of ceramic nanopowders: (**a**) s-1.67, (**b**) c-1.67, (**c**) 1.5, and (**d**) 1.0.

Figure 3 exhibits the XRD patterns of the ceramic nanopowders tested. The diffractograms of s-1.67 and c-1.67, according to International Center for Diffraction Data File Card No. 01-080-7085, were assigned to the hexagonal structure of hydroxyapatite (P63/m space group). Both samples s-1.67 and c-1.67 show the peak with the highest intensity at 31.74° of two theta (211), assigned which corresponds to the main peak of the HA hexagonal structure. The XRD results obtained demonstrate that the developed hydroxyapatite synthesis method enables the preparation of crystalline and phase-pure hydroxyapatite [50,53].

Based on the analysis of the diffractogram of the sample labeled 1.5, the highest intensity is shown by the peak at 31.02° of two theta (0210), which is characteristic of the rhombohedral structure of β-TCP (R3c space group) [54]. The XRD result was found to be consistent with the phases listed in the ICDD database File Card No. 04-008-8714 assigned to β-TCP.

The reflections obtained for the sample designated 1.0 in the XRD study show characteristic peaks assigned to the monoclinic structure of brushite (Ia space group). Analysis of the obtained diffractogram shows characteristic peaks at two theta equal to 11.60° (020) and 23.39° (040), which are in accordance with the File Card No. 00-011-0293 in the ICDD database.

The degree of crystallinity (*X_c_*) of samples was calculated using OriginPro 2023 software. The crystalline fraction was determined by integrating the areas under the crystalline (*P_c_*) and amorphous (*P_a_*) diffractions pattern and calculated in accordance with the following formula:

$$X_{c}=\frac{P_{c}}{P_{a}+P_{c}}$$

The obtained results have shown significant differences in crystallinity among samples. The degree of crystallinity of s-1.67 and c-1.67 were 41.5% and 48.6%, respectively. On the other hand, the highest crystallinity was observed for sample 1.5 (82.8%), whereas the crystallinity degree of sample 1.0 was 75.1%. On this basis, it can be concluded that the method produces phase-pure and crystalline [55,56].

### 3.3. Fourier Transform Infrared Spectroscopy Analysis

**Figure 4:**
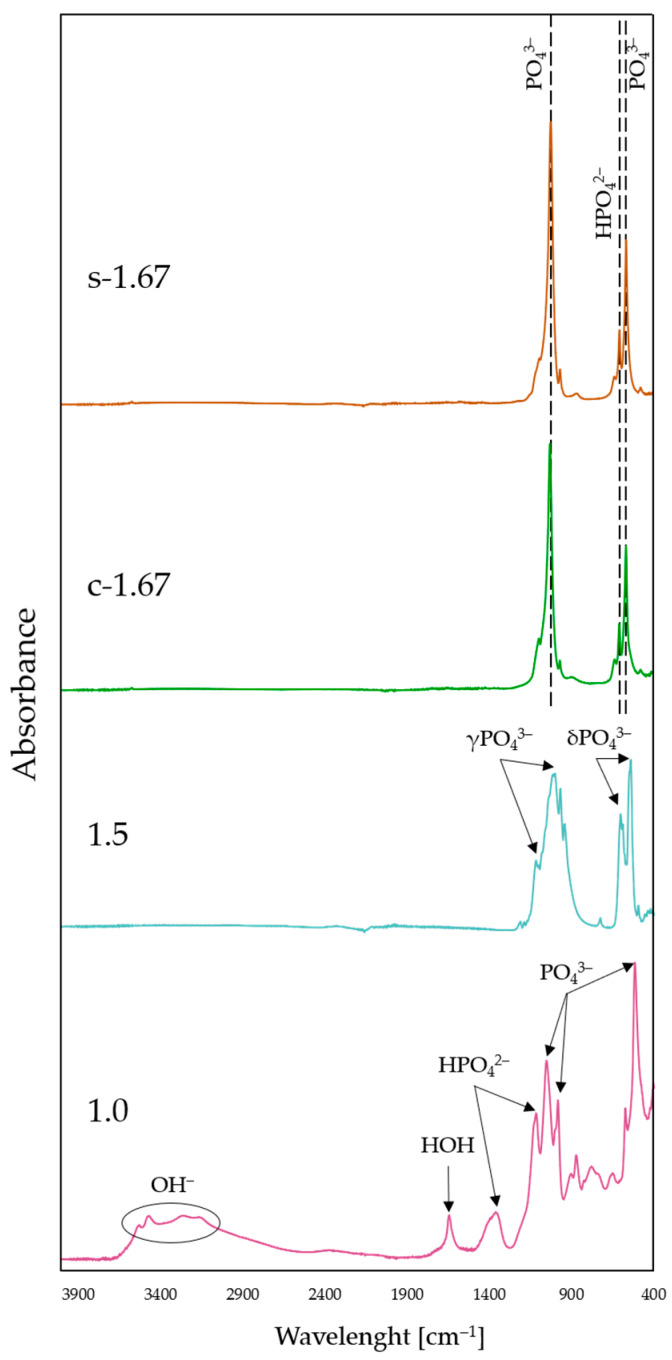
FT-IR spectra of nanopowders: s-1.67, c-1.67, 1.5, and 1.0.

The comparative analysis of the FT-IR spectra of the ceramic nanopowders is shown in Figure 4. Both the spectrum of sample s-1.67 and c-1.67 show characteristic peaks for hydroxyapatite. The spectrum at 1018 cm^−1^ can be identified as asymmetric P–O stretching vibrations. The peaks at 559 cm^−1^ and 599 cm^−1^ have been attributed to triple degenerate O–P–O stretching modes in PO_4_^3−^. The peaks of the characteristic functional groups present in the synthesized hydroxyapatite overlap with the FT-IR spectrum of commercial hydroxyapatite [47,57].

On the FT-IR spectrum for the sample marked 1.5, peaks at a wavenumber of 541 cm^−1^ and 603 cm^−1^, attributed to triply degenerated asymmetric bending vibrations for PO_4_^3−^, are visible. Spectral analysis revealed the presence of triply degenerated asymmetric stretching of the orthophosphate groups of PO_4_^3−^, as evidenced by distinct bands in the range 1015–1116 cm^−1^. Furthermore, at 944 cm^−1^ and 979 cm^−1^, symmetric stretching is also attributed to the PO_4_^3−^ group. The FT-IR spectral analysis performed indicates that the phase obtained was β-TCP [54,58,59,60].

FT-IR spectral analysis of sample 1.0 indicates that the vibrations at 1118 and 1203 cm^−1^, which are characteristic of the brushite phase, can be attributed to the monohydrogen phosphate ion HPO_4_^2−^. The peaks at 572 cm^−1^, 983 cm^−1^ and 1052 cm^−1^ correspond to the PO_4_^3−^ group. Furthermore, the spectral band occurring between 3153 and 3532 cm^−1^ is attributed to the OH^-^ group. Moreover, the characteristic peak at 1645 cm^−1^ is assigned to HOH [56,61,62,63].

Based on the FT-IR spectral analysis of the ceramic nanopowders, the presence of characteristic functional groups was identified, and consequently, it was confirmed that monophasic compounds are obtained as a result of the developed methods for the synthesis of calcium phosphate ceramics.

### 3.4. Specific Surface Area and Porosity Studies

Importantly, the morphological properties such as high surface area, large pore volume, and narrow particle size distribution are well suited for the application of nano-encapsulation in drug delivery systems. The results obtained are presented in Table 3.

**Figure 5:**
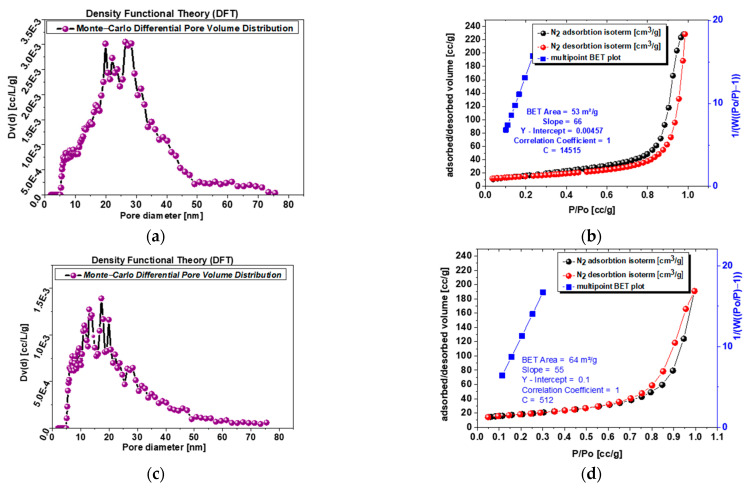

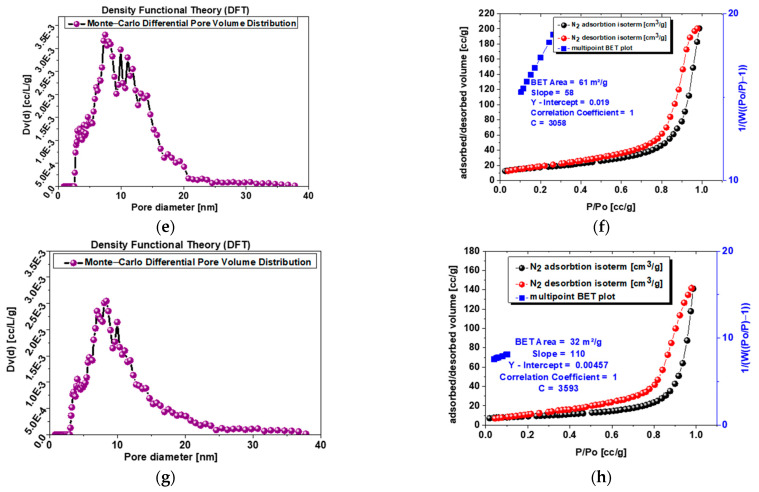
(**a**) Sample s-1.67 mesopore size differential distribution according to DFT (Dv(d) [cc/L/g]; (**b**) sample s-1.67 adsorption–desorption isotherms and multipoint BET plot; (**c**) sample c-1.67 mesopore size differential distribution according to DFT (Dv(d) [cc/L/g]; (**d**) sample c-1.67 adsorption–desorption isotherms and multipoint BET plot; (**e**) sample 1.5 mesopore size differential distribution according to DFT (Dv(d) [cc/L/g]l; (**f**) sample 1.5 adsorption–desorption isotherms and multipoint BET plot.; (**g**) sample 1.0 mesopore size differential distribution according to DFT (Dv(d) [cc/L/g]; (**h**) sample 1.0 adsorption–desorption isotherms and multipoint BET plot.

Experimental adsorption and desorption isotherms of nitrogen (77 K) are presented in Figure 5b,d,f,h. The investigated powders 1.0 and s-1.67 reveal mesoporosity and microporosity structure, while samples 1.5 and c-1.67 reveal mesopore structure, while microporosity is 0 for both samples. This is supported by the shape of the isotherms, which are of type IV (according to IUPAC classification). The specific surface area is related to mesoporosity changes from 64 to 61 m^3^/g for c-1.67 and powder 1.5 to 53–32 m^3^/g for powder s-1.67 and sample 1.0. The higher the specific surface area, the better drug delivery systems can be developed on the basis of this powder.

Analysis of Figure 5a,c,e,g with pore volume distribution profiles exhibit single narrow peaks for sample 1.0 and powder s-1.67 with a maximum at 8–12 nm, while powder 1.5 shows more broad peaks at a similar range of 8–13 nm, and the most blurred peaks at widest range represents commercial sample c-1.67 at 15–30 m.

### 3.5. Kinetic Release Studies of Antibiotic

**Figure 6:**
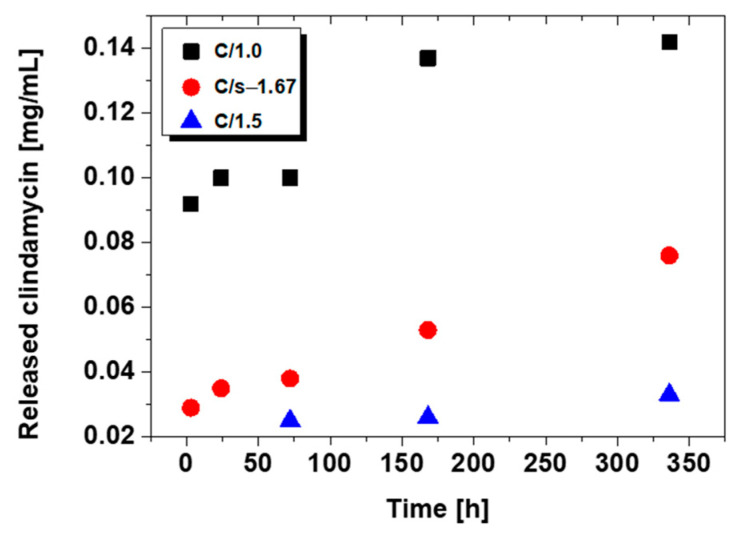
Release profile of clindamycin from modified ceramic powders.

The amount of clindamycin in mg/mL released from the powder samples is presented in Figure 6.

The release results for C/1.0, C/1.5, and C/s-1.67 are presented. For commercial hydroxyapatite C/c-1.67, the amount of drug released was too small to be determined. As can be observed, after a 14-day immersion, the largest amount of drug is released from brushite (C/1.0), and the amount is practically double that for synthetic hydroxyapatite (C/s-1.67). In the case of TCP (C/1.5), clindamycin was only able to be determined on day three after immersion in PBS.

**Figure 7:**
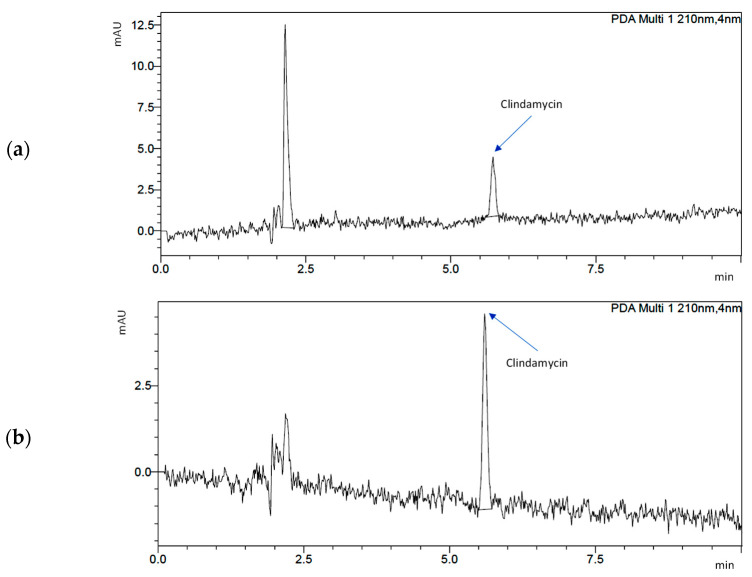
Chromatograms demonstrating drug release from s-1.67: (**a**) after 1 day of incubation and (**b**) after 14 days of incubation.

Chromatograms for C/s-1.67 powder (after 24 h and 14 days) are presented in Figure 7. The peak for clindamycin was determined at retention times of 5727 (day 1) and 5604 (day 14). The results indicate that the release of clindamycin is dependent on time and type of powder. It was indicated that during incubation, clindamycin content in the liquid increased continuously for 14 days. It was also observed that the smaller the specific surface area of the powder, the faster the drug is released (see Section 3.4 Specific Surface Area and Porosity Studies).

Finally, it is important to clarify the amount of drug selected for powder modification, a solution with a concentration of 2 mg/mL. The recommended therapeutic dose of clindamycin for an adult (about 70 kg) starts at 150 mg. However, this is the conventional amount that should be taken orally. This means that a sizable portion of the active ingredient will already be degraded in the gastrointestinal tract, and in addition, much of it will undergo general distribution throughout the body. As a result, only a small part of the drug will reach the affected area. Drug carriers being developed for targeted therapy allow the active ingredient to act directly at the site where the therapeutic effect is expected. This involves a significant reduction in the amount of antibiotic the patient should receive. The dose can be increased or decreased depending on individual needs.

### 3.6. Morphology Analysis

**Figure 8:**
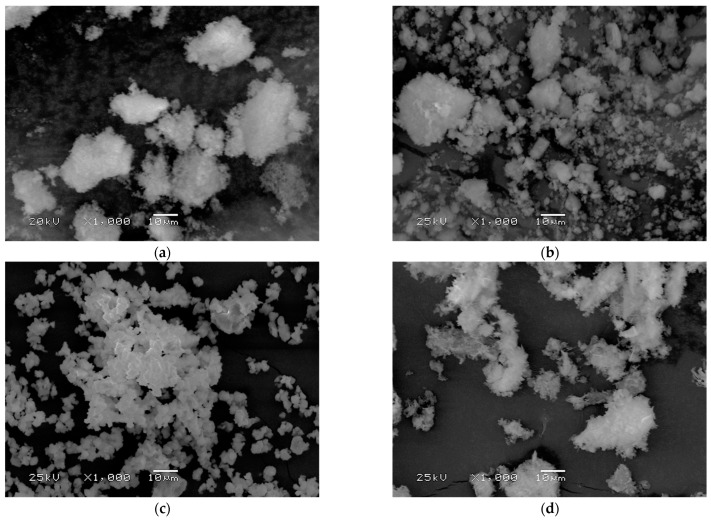
Powder surface morphology for (**a**) s-1.67, (**b**) c-1.67, (**c**) 1.5, and (**d**) 1.0.

Scanning electron microscopy imaging was carried out to determine the grain shape of each powder (Figure 8). A high similarity was observed between s-1.67 and 1.0. In both cases, the powder took on a flocculent shape in small agglomerates. The flocculent structure can also be seen in the case of commercial hydroxyapatite c-1.67. The most regular, granular shape was observed for TCP 1.5.

**Figure 9:**
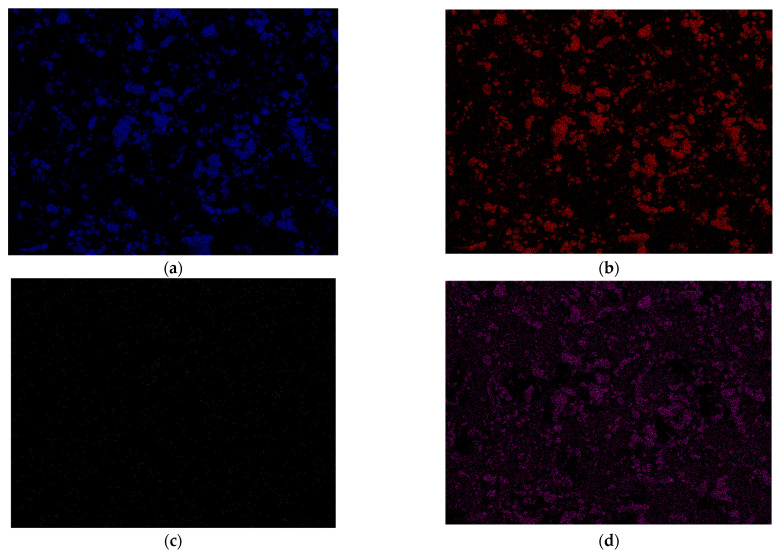
EDS microanalysis for C/s-1.67. Determination of elements (**a**) Ca, (**b**) P, (**c**) Cl, and (**d**) Au.

Using EDS microanalysis, the presence of individual elements in C-s-1.67 was determined (Figure 9). Au was measured considering the necessity of sputtering the surface of the samples with gold. Ca and P because these are the main components of the calcium phosphates prepared within this publication. Cl, on the other hand, was determined, in a manner of speaking, to validate the presence of the antibiotic in the modified powder. The molecular formula of clindamycin hydrochloride, as was used during this study, is C_18_H_34_Cl_2_N_2_O_5_S. Hence, if chlorine ions are present on the EDS mapping, it means that they come directly from the bound drug.

In the case of Ca and P (blue and red colors), overlapping elemental maps were observed. This is due to the connections between these elements in the structure of calcium phosphates. Small green signals from chlorine were observed, distributed fairly parallel across the entire volume of the test powder. This suggests an effective drug modification and confirms the presence of the antibiotic in the ceramics.

### 3.7. Antimicrobial Activity of Clindamycin-Modified Ceramic Powders

It was demonstrated that all tested ceramic powders containing clindamycin exhibited strong antibacterial activity towards *Staphylococcus aureus*, which gives evidence that the antibiotic stayed active and in the range of biologically effective concentrations. Whereas materials lacking this antibiotic did not exhibit antimicrobial activity. The MIC values (Table 4) for the C/s-1.67 and C/1.0 ceramic powders were 12.5 µg/mL, which is 100-fold higher than the MIC value of clindamycin, while the C/1.5 ceramic powder showed a higher MIC value equal to 25 µg/mL, and the highest MIC (weakest antimicrobial potential) was observed for the C/c-1.67 ceramic powder. The MIC value for the reference antibiotic, clindamycin, was 0.125 ug/mL, which confirms the susceptibility of the selected strain to clindamycin [52].

**Figure 10:**
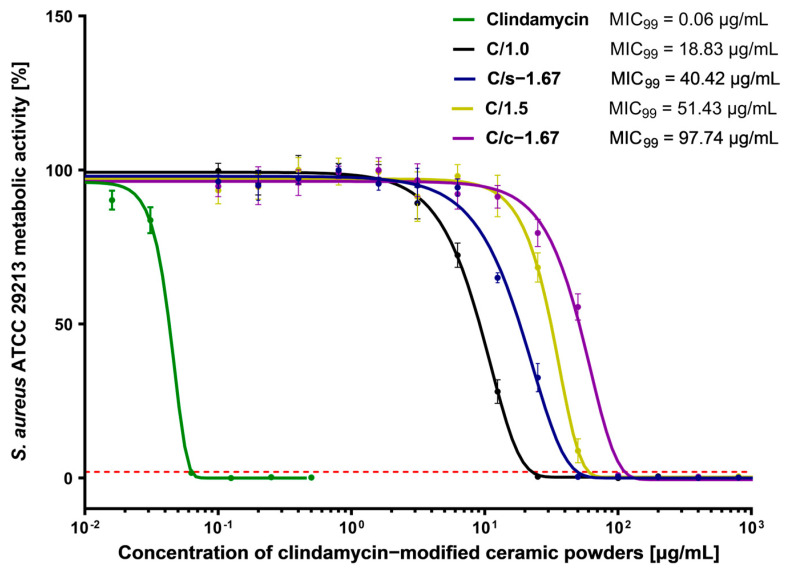
Dose-response curves of clindamycin-modified ceramic powders against *Staphylococcus aureus* ATCC 29213 and MIC_99_ values. The green line represents the reference curve of bacterial metabolic inhibition for clindamycin. The intersection of the dose-effect curves with the red line (the 99% reduction in bacterial metabolic activity) is identified as the MIC_99_ value. Each point represents the averages of four independent experiments in triplicate. Error bars indicate standard deviations.

To address the question of whether the obtained clindamycin-modified powders exhibit antibacterial activity, we have also evaluated MIC_99_ values, defined as a concentration that causes a 99% reduction in the metabolic activity of bacterial cells. Resazurin reduction assay is reported as a non-cytotoxic assay to monitor the metabolic activity and proliferation of live bacterial cells. Resazurin, a non-fluorescent water-soluble dye, is reduced to highly fluorescent resorufin in proportion to the metabolic activity of a bacterial cell population [51]. As illustrated in Figure 10, the lowest MIC_99_ value for all tested clindamycin-loaded ceramics was shown for C/1.0 powder (18.83 ± 1.23 μg/mL), which indicates the strongest antimicrobial activity. We have shown that C/s-1.67 (40.42 ± 5.80 μg/mL) had significantly higher antimicrobial properties compared to the C/c-1.67 (97.74 ± 15.50 μg/mL).

Statistical analyses and graphs for biological studies were performed using GraphPad Prism version 9.1.0 for Windows (GraphPad Software, San Diego, CA, USA). Data were compared using one-way ANOVA with Dunnett’s post hoc test.

## 4. Discussion

In conclusion, ceramic materials with different morphologies have been successfully synthesized by wet precipitation methods. A number of analyses carried out allow us to conclude that the selected methods of synthesis by the wet precipitation technique make it possible to obtain ceramic powders of TCP, brushite, and hydroxyapatite. This fact was confirmed by FTIR analysis, where the main functional groups present in the materials were assigned, as well as by XRD analysis, in which the obtained diffractograms were related to the corresponding data sheets. All powders were phase-pure; however, the observed background spectrum suggests that they are only partially crystalline and partially amorphous. In addition, hydroxyapatite s-1.67 was compared with its commercial counterpart, c-1.67. Presumably, high-temperature calcination would enable materials with a higher degree of crystallinity to be obtained. This study also confirmed that based on the same substrates, changing only their starting ratios and not changing the process conditions, it is possible to obtain both synthetic hydroxyapatite s-1.67 and TCP 1.5. This is an important fact considering the different properties of the two materials and their potential applications.

The chosen physical sorption method was successful in modifying ceramic powders. This is evidenced by the presence of chlorine ions derived from the drug as detected by SEM-EDS. However, it can be concluded that the materials, especially the commercial c-1.67, do not show high clindamycin loading efficiency. A relationship was observed between the specific surface area of the ceramic and drug release. The smaller the specific surface area, the faster the drug is released. This is in accordance with literature reports, as a larger pore surface area would result in a more efficient migration of the drug into the interior of the ceramic and, thus, a later, longer drug release [64]. Drug adsorption on ceramic powders is mainly accomplished by forming bonds between Ca^2+^ ions on the ceramic surface and oxygen atoms in the drug molecule [65]. This study exhibits that the degree of crystallinity of the tested nanopowders influences the loading capacity of the active substance, as well as the amount of drug released. For sample 1.0, with a degree of crystallinity of 75.1%, the highest concentration of clindamycin was obtained. However, a different correlation was observed for sample 1.5, where the degree of crystallinity is the highest, while less drug was released.

Determining the pore size distribution, the smallest pores observed were for brushite, a ceramic with a Ca/P ratio of 1.0, and synthetic hydroxyapatite s-1.67. A biological correlation was observed for these powders, as these two powders demonstrated the highest antimicrobial ability. Naturally, pure clindamycin exhibited the greatest antimicrobial ability against *Staphylococcus aureus*, while the ceramic powders themselves not modified with the drug did not demonstrate it at all. A close correlation was observed between the results obtained for the biological tests on antimicrobial capacity and the results for drug release from HPLC.

The studies presented in this manuscript confirm the effect of specific surface area on the nature of modification of porous powders by physical sorption, as well as the subsequent rate of drug release. Considering that the s-1.67 and 1.5 powders were based on the same method, the same conditions were maintained during synthesis, and only the concentration of initial substrates differed, it suggests that this aspect also affects the size of the specific surface area since a slight difference was observed between the values for these powders. However, it can be concluded that ceramic porous materials have been successfully synthesized as a drug carrier for targeted therapy.

## Figures and Tables

**Table 1:** Synthetic calcium phosphates relevant to medical applications.

Calcium Phosphates in the CaO-P_2_O_5_ Arrangement				
Abbreviation	Systematic Name	Mineralogical Name	Chemical Formula	Molar Ratio Ca/P
CP	Calcium metaphosphate	–	Ca(PO_3_)_2_	0.5
C_2_P	Calcium pyrophosphate	–	Ca_2_P_2_O_7_	1.0
C_3_P, TCP	Tricalcium phospahte	Whitlockite	Ca_3_(PO_4_)_2_	1.5
C_4_P, TTCP	Tetracalcium phosphate	–	Ca_4_(PO_4_)_2_O	2.0
**Calcium Phosphates in the CaO-P_2_O_5_-H_2_O Arrangement**				
**Abbreviation**	**Systematic Name**	**Mineralogical Name**	**Chemical** **Formula**	**Molar Ratio Ca/P**
MCPA	Calcium dihydrogenphosphate	–	Ca(H_2_PO4)_2_	0.5
MCPM	Calcium dihydrogenphosphate monohydrate	–	Ca(H_2_PO_4_)_2_ ⋅H_2_O	0.5
DCPD	Dicalcium phosphate dihydrate	Brushite	CaHPO_4_ ⋅2 H_2_O	1.0
DCPA	Dicalcium phosphate anhydrous	Monethite	CaHPO_4_	1.0
OCP	Octacalcium phosphate	–	Ca_8_H_2_(PO_4_)_6_ ⋅5 H_2_O	1.333
HA, HAp, OHAp	Pentacalcium hydroxide triphosphate	Hydroxyapatite	Ca_10_(PO_4_)_6_ ⋅2 H_2_O	1.667

**Table 2:** Calcium and phosphorus content and Ca/P molar ratio in powders (mean ± SD).

Sample	Ca Content (wt. %)	P Content (wt. %)	Ca/P Molar Ratio
s-1.67	37.51 ± 0.65	17.26 ± 0.19	1.67
c-1.67	37.46 ± 0.38	17.27 ± 0.12	1.67
1.5	36.93 ± 0.32	18.87 ± 0.23	1.51
1.0	37.81 ± 0.41	27.91 ± 0.34	1.04

**Table 3:** N2 physisorption-derived parameters characterizing the obtained samples (SSA—specific surface area).

Sample	SSA [m^2^/g]	Porosity		
		Pore Size Distribution DFT [nm]	Pore Volume Macropores [cm^3^/g]	Pore Area Micropores Vt [m^2^/g]
s-1.67	53	4, 10, 11, 13	0.345	3.52
c-1.67	64	11, 13, 17, 20, 22, 24, 28	0.27	0
1.5	61	8, 10, 11, 13	0.304	0
1.0	32	4, 7, 8, 10	0.21	4.94

**Table 4:** Minimum inhibitory concentration (MIC) of ceramic powders against *Staphylococcus aureus*.

Ceramic Powder	MIC [µg/mL]
s-1.67	>25,600
C/s-1.67	12.5
c-1.67	>25,600
C/c-1.67	50
1.5	>25,600
C/1.5	25
1.0	>25,600
C/1.0	12.5
Clindamycin	0.125

## Data Availability

Data that support the findings of this study are contained within the article.
